# Transformation and regeneration of the holoparasitic plant *Phelipanche aegyptiaca*

**DOI:** 10.1186/1746-4811-7-36

**Published:** 2011-11-08

**Authors:** Mónica Fernández-Aparicio, Diego Rubiales, Pradeepa CG Bandaranayake, John I Yoder, James H Westwood

**Affiliations:** 1 Institute for Sustainable Agriculture, IAS-CSIC, Dept. of Plant Breeding, Córdoba, 14080, Spain; 2 Virginia Tech, Dept. of Plant Pathology, Physiology and Weed Science, Blacksburg, VA 24061, USA; 3 University of California-Davis, Dept. of Plant Sciences, Davis, CA 95616, USA

**Keywords:** holoparasitic plants, gene transformation, haustorium, *Phelipanche*, *Orobanche*

## Abstract

**Background:**

Transformation and subsequent regeneration of holoparasitic plants has never been reported, in part due to challenges in developing transformation protocols, but also because regeneration of obligate parasites is difficult since their survival depends completely on successful haustorium penetration of a host and the formation of vascular connections. The recent completion of a massive transcriptome sequencing project (the Parasitic Plant Genome Project) will fuel the use of genomic tools for studies on parasitic plants. A reliable system for holoparasite transformation is needed to realize the full value of this resource for reverse genetics and functional genomics studies.

**Results:**

Here we demonstrate that transformation of *Phelipanche aegyptiaca *is achieved by infection of 3 month-old *in vitro *grown *P. aegyptiaca *calli with *Agrobacterium rhizogenes *harboring the yellow fluorescent protein (YFP). Four months later, YFP-positive regenerated calli were inoculated onto tomato plants growing in a minirhizotron system. Eight days after inoculation, transgenic parasite tissue formed lateral haustoria that penetrated the host and could be visualized under UV illumination through intact host root tissue. YFP-positive shoot buds were observed one month after inoculation.

**Conclusions:**

This work constitutes a breakthrough in holoparasitic plant research methods. The method described here is a robust system for transformation and regeneration of a holoparasitic plant and will facilitate research on unique parasitic plant capabilities such as host plant recognition, haustorial formation, penetration and vascular connection.

## Background

Parasitic weeds belonging to the genera *Orobanche *and *Phelipanche *have lost through evolution their autotrophic way of life, switching from photosynthesis to obtaining their resources by parasitizing other plants. Parasites capture host water and nutrients through a specialized organ, the haustorium, which invades the host root and connects with the host vascular system [1-3]. *Phelipanche aegyptiaca *(syn. *Orobanche aegyptiaca*) is an important parasitic weed attacking many crops in Asia and the Middle East. Conventional control based on cultural methods, herbicides, or host breeding for parasitic plant resistance, have not attained complete success due to several factors associated with the parasite life cycle, including high fecundity (hundreds of thousands of seeds per parasite), seed longevity in the soil, the subterranean location of the young parasite that effectively hides it from the farmer, the tightly coordinated parasitic and host life cycles, and the scarcity of sources of resistance in most affected crop species [4-6]. Biotechnological control approaches based on crops harboring transgenic resistance to the parasite, resistance to herbicides, or expressing silencing constructs targeting genes essential to an attached parasite remain rare, but have shown promise for protecting crops [7,8].

Progress in understanding the unique biology of *Orobanche *and *Phelipanche *spp. has been hampered by a lack of genomic resources and transformation protocols for these parasites. A solution to the former problem has recently emerged through the Parasitic Plant Genome Project, which has produced more than 1.3 billion ESTs expressed at various life stages of the parasitic plants *Triphysaria versicolor*, *Striga hermonthica *and *P. aegyptiaca *[9]. This is a major advance, but reverse genetics will be essential to discover the roles of these sequences in the parasitic phenotype and to be able to assign functions to parasite genes [10]. To our knowledge there are no previous reports describing the development of a transformation system for any holoparasitic species. There is one report in which *P. aegyptiaca *gene expression was manipulated via hairpin RNAi targeted to the mannose 6-phosphate reductase gene. However, this construct acted indirectly through a transformed tomato host and relied on the systemic movement of the silencing signal through the haustorial connection, so no *Orobanche *transformation ocurred [11]. Transformation has been accomplished in the facultative parasites *T. versicolor *[12] and *Phtheirospermum japonicum *[13], and the former has proved useful in partially dissecting the haustorium signal transduction pathway [14].

The great challenge in generating transformed holoparasitic plants is in recovering a high percentage of transformants in a species for which the key step - successful establishement on a host - is a low frequency event. For *Orobanche *and *Phelipanche *seedlings, once germination is induced by host root-exuded stimulants [15,16], survival depends on the ability to connect to the host or else perish after several days without nutritional supply from a host [17]. In the field this short independence phase lasts for just a few days after germination, but under laboratory conditions it is possible to prolong independence for months by cultivating parasite callus in nutritive media [18,19]. However studies based on *in vitro *growth of *Orobanche *and *Phelipanche *species have been considered difficult because the parasite requires a host for normal development [20]. Here we report the development of a simple and efficient method for *Agrobacterium rhizogenes*-mediated transformation and subsequent regeneration by using cultured callus as starting tissue for both transformation and regeneration.

## Results and Discussion

### *P. aegyptiaca in vitro *growth

**Figure 1:**
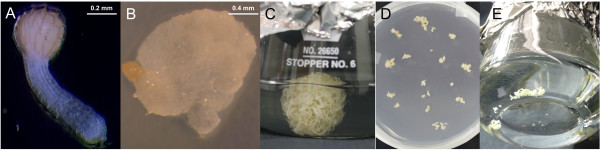
***P. aegyptiaca *in vitro sterile conditions for growth and transformation**. A) *P. aegyptiaca *radicle emerged from the seed coat 7 days after GR24 application. B) One-month-old *P. aegyptiaca *callus developed from seedling with seed coat still visible. C) Three-month-old *P. aegyptiaca *callus just prior to transformation. D) Fragments of the *P. aegyptiaca *callus chopped into multiple small explants and co-cultivated in the dark with *A. rhizogenes *on solid media. E) *P. aegyptiaca *explants returned to liquid media 5 days after transformation.

Holoparasitic plants from the *Orobanche *and *Phelipanche *genera need exposure to exogenous stimulants exuded by host roots in order to germinate [15,16]. Germination of *P. aegyptiaca *seeds was induced in this work by application of the synthetic germination stimulant GR24 [21]. After 24 hours of GR24 exposure, seeds were transferred to MS medium supplemented with sucrose. Germination occurred from three to seven days after GR24 application (Figure 1A). During the following days the parasite radicles on MS medium grew in length and width, and 30 days after GR24 addition, 80 ± 2.3% of the parasitic radicles had developed tubercle-like swelling to a diameter ≥ 2.0 mm (Figure 1B). Some of the radicles did not develop into a tubercle-like structure, remaining unchanged, neither dying nor developing, while other neighbouring seedling radicles rapidly developed into healthy callus. This observation could point to a *Phelipanche *genotypic effect on the ability of a seedling radicle to develop into a callus under *in vitro *conditions. Subsequently, the healthiest calli, as indicated by large size, white color (illustrated in Figure 1B) were selected for transfer to liquid media. These calli, growing in full strength MS liquid medium showed rapid growth and after two months they presented a compact texture with a diameter of approximately 2 cm and exhibiting initiation of multiple peripheral roots (Figure 1C), which were then used for *A. rhizogenes *inoculation.

### Development of *Agrobacterium*-mediated *P. aegyptiaca *transformation

**Figure 2:**
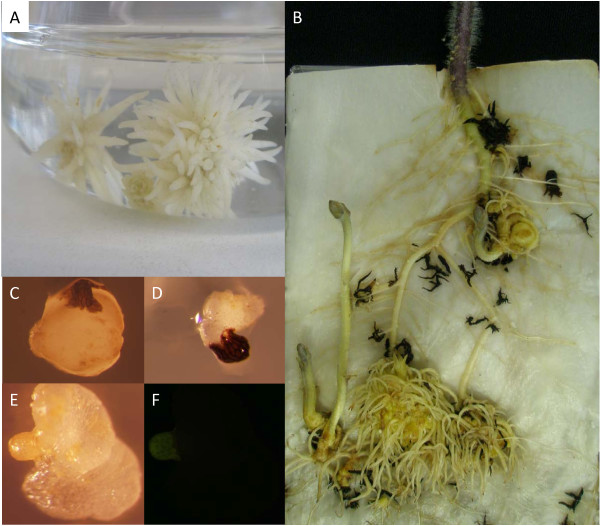
**Selection and regeneration of *P. aegyptiaca***. A) *P. aegyptiaca *clones with parasitic infective roots ready to be inoculated on tomato. B) Sucessful *P. aegyptiaca *explants infecting tomato host root in a minirhizotron system, demonstrating that the parasite is able to regenerate its whole plant structure, including anchorage roots and shoots. Black explants were not able to infect the tomato roots. C) Wild type *P. aegyptiaca *callus growing in MS media without kanamycin. D) Wild type *P. aegyptiaca *callus growing in MS media suplemented with 100 μg/ml of kanamycin. E) Wild type *P. aegyptiaca *callus observed under white light and F) UV illuminated image of E, showing only slight autofluorescence from the seed coat.

As a first step in designing a selection and regeneration strategy for *P. aegyptiaca *it was necessary to determine the callus growth capacity and response to antibiotics and UV illumination. For regeneration, *P. aegyptiaca *calli growing in MS liquid media were divided into multiple small pieces with a scalpel and returned to liquid medium. These explants comprised in a population of clones (Figure 2A) that were inoculated onto tomato roots and which showed infectivity potential by attaching to hosts and developing to the point of shoot production (Figure 2B). To identify an optimal selective agent, one-month-old *P. aegyptiaca *calli were grown on media containing kanamycin. Ten wild-type *P. aegyptiaca *calli were grown in MS medium supplemented with a range of kanamycin concentrations from 0 μg/ml (control) (Figure 2C), to 50 μg/ml and 100 μg/ml (Figure 2D). Kanamycin did not cause death of the wild-type parasitic calli, although growth of *P. aegyptiaca *was slightly reduced at the highest kanamycin concentration. As an alternative to antibiotic selection, the feasibility of fluorescent markers was evaluated by observing parasites under UV light and 23 day-old *P. aegyptiaca *wild-type calli showed no autofluorescence except for the seed coat (Figures 2E and 2F). Therefore a fluorescent marker was determined to be more reliable than kanamycin resistance for screaning transgenic tissue.

**Figure 3:**
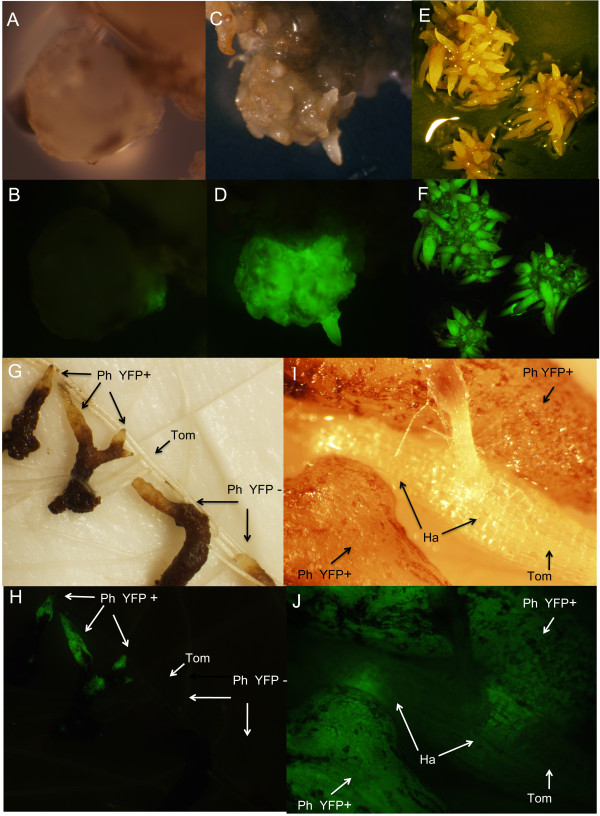
**Selection and regeneration of transformed callus**. White light (A, C, E, G, and I) and UV illuminated (B, D, F, H, and J) *P. aegyptiaca *tissues showing YFP fluorescence. A and B) *in vitro *callus of *P. aegyptiaca *expressing a small sector of YFP on day 35 after transformation. C and D) chimeric transgenic *P. aegyptiaca *on day 90 after transformation. YFP-negative tissue was cut away and the YFP- positive tissue subjected to clonal propagation resulting in homogeneus transgenic calli seen in E and F. E and F) clonally propagated calli used for tomato inoculation on day 120 after transformation. G and H) tomato inoculation with excised explants from the transgenic calli. YFP-positive and negative parasitic roots were inoculated next to each other to show the absence of fluorescence in tomato roots and YFP-negative explants. I and J) Detail image of tomato host root parasitized by *P. aegyptiaca *explants from both sides. The wedge-shaped haustoria penetrating the host root are visible through the intact tomato living tissue. Ph YFP+, *P. aegyptiaca *positive expressing YFP; Ph YFP-, *P. aegyptiaca *negative control; Tom, tomato root; Ha, YFP positive haustoria.

One *P. aegyptiaca *callus of high quality (e.g., Figure 1C) was choosen and divided into more than 100 small pieces with average diameters of 5 mm at the time of agroinfection. This allows for a rapid wound-mediated inoculation of *Agrobacterium *into the *P. aegyptiaca *tissue. Furthermore, in this way the transgenic *P. aegyptiaca *individuals obtained from any transformation events would be clones, sharing the same genotype but only differing in the gene transfection event. This allows a more accurate evaluation of the gene effect on phenotype in future work. *A. rhizogenes *strain MSU440 [22] carried the binary vector pHG8-YFP modified from pHellsgate8 by cloning into the SacI site the mas-YFP fusion for visual screening of transgenic *P. aegyptiaca *tissue [14]. Thirty-five days after *A. rhizogenes *inoculation, 15% of explants showed a sector of YFP fluorescence (Figures 3A and 3B). The transformation frequency largely depends on the transformable species in study, but no previous reports exist for *Orobanche or Phelipanche *species. In *T. versicolor*, agroinfection resulted in a transformation frequency of up to 8.3% [12] and was later optimized to achieve a transformation frequency up to 33% [14].

**Figure 4:**
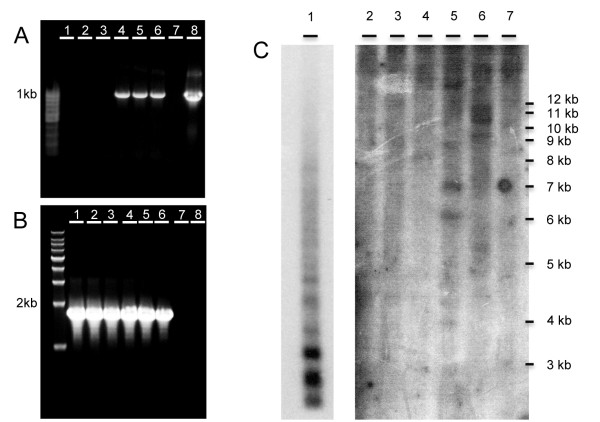
**Detection of YFP reporter gene by PCR and Southern blot hybridization**. A) Amplification of mas-YFP sequence from genomic DNA of non transgenic (lanes 1-3) and transgenic (lanes 4-6) *P. aegyptiaca *cultures; lane 7, ntc; lane 8, plasmid pHG8-YFP. B) Amplification of 18S from the same non transgenic (lane 1-3) and transgenic (lane 4-6) genomic DNA used in panel A; lane 7, ntc; lane 8, plasmid pHG8-YFP. C) Southern blot hybridization of mas-YFP probe to plasmid pHG8-YFP (lane 1, 1 hour exposure). Genomic DNA of non transgenic *P. aegyptiaca *callus digested with EcoR1 (Lane 2), EcoRV (Lane 3), XbaI (Lane 4) and YFP- expressing *P. aegyptiaca *callus digested with EcoR1 (Lane 5), EcoRV (Lane 6), and XbaI (Lane 7). Image for lanes 2-7 was 9 days exposure after after removing lane 1.

The YFP-positive explants were grown for an additional two months and monitored for development of YFP-positive tissues, which formed parts of chimeric calli 90 days after transformation (Figures 3C and 3D). In order to obtain pure transgenic calli, the YFP-negative regions were cut away, leaving only the YFP-positive tissue (Figures 3E and 3F). A similar strategy was used by Bandaranayake et al. [14] in which YFP-negative roots were excised with a scalpel from transgenic autotrophic *Triphysaria *plants. To confirm that calli were transgenic, a fragment of approximately 1000 bp of the mas-YFP reporter construct was amplified by PCR from fluorescent calli, while non-fluorescent control calli yielded no corresponding PCR product. The same product was also amplified in the positive control reaction using the plasmid pHG8-YFP as a template while no product was observed in the negative reaction (Figure 4A). To verify template quality a separate PCR was used to amplify a 1800 bp fragment of the 18S gene, and a product was produced in both fluorescent and non fluorescent control calli (Figure 4B). Evidence for genomic integration of the transgene was provided by a Southern blot hybridization using a probe homologous to the mas-YFP sequence. Genomic DNA from non-fluorescing and fluorescing calli was digested with three restriction enzymes, fragments separated, and probed. DNA from the YFP positive calli produced multiple bands (Figure 4C, lanes 5-7) that were not apparent in the control lanes (Figure 4C, lanes 2-4). The restriction enzymes were predicted to not cut in the probe region, so the bands in lanes 5 and 6 suggest the presence of four insertion sites.

### Clonal propagation and regeneration of transgenic *Phelipanche*

The *Phelipanche *transformation method takes advantage of the ability to asexualy propagate *Phelipanche *such that multiple calli can be generated for each transformation event (Figures 3E and 3F). This clonal propagation step is a novel feature of our protocol and serves the dual function of producing sufficient material for direct assays of parasite infection via lateral haustoria and greatly enhancing chances of successfully inoculationg a host and completing the full life cycle of a transformed plant. *Phelipanche in vitro *calli that developed from individual seeds were previously reported to be infective on host roots growing in aseptic conditions, but the percentage of successful infections was low [23]. Subsequently this method was improved by Zhou et al. [20] using different media and hormonal supplementation. A remarkable feature of our protocol is that it avoids the use of any hormonal supplementation, which simplifies both the procedure itself and subsequent analyses of tubercle gene expression by eliminating potentially confounding hormonal interactions with parasite gene expression and development.

**Figure 5:**
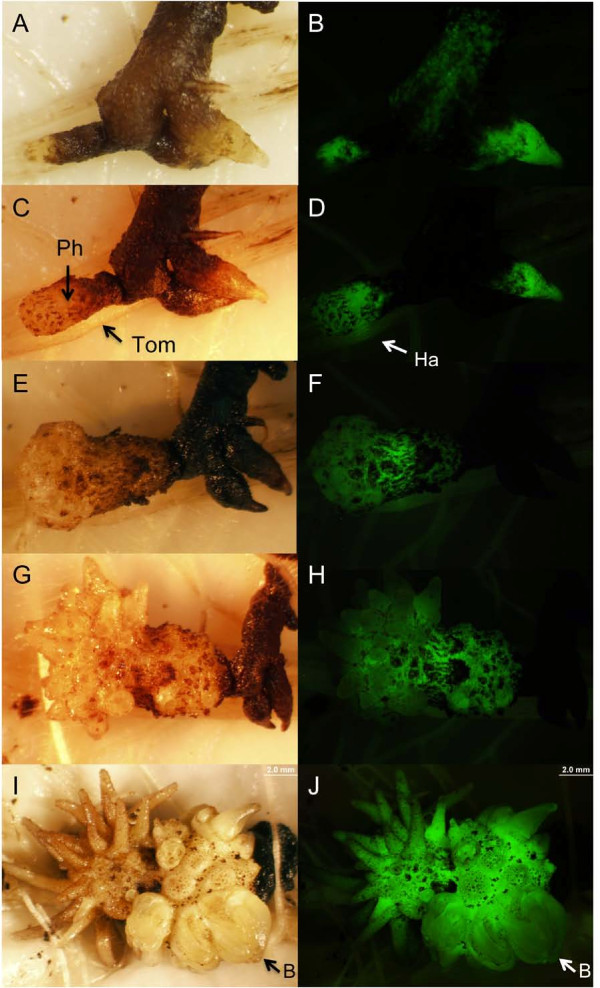
**Regeneration of transgenic *P. aegyptiaca *by inoculation onto tomato host roots**. White light (A, C, E, G, and I) and UV illumination (B, D, F, H, and J) of transgenic *P. aegyptiaca*. A and B) Explants 4 days after inoculation onto tomato roots. C and D) Eight days after inoculation *P. aegyptiaca *developed a haustorium and penetrated the tomato root. E and F) Twenty days after inoculation the parasite established full vascular connection with the tomato host, developing a tubercle near the point of haustorial attachment. The rest of inoculated parasitic explant tissue has died. G and H) Twenty five days after inoculation the *P. aegyptiaca *transgenic tubercle developed the spider-like stage. I and J) Thirty days after inoculation transgenic parasitic shoot buds regenerated. Ph, *P. aegyptiaca*; Tom, tomato; Ha, haustorium; B, shoot bud.

To demonstrate infectivity of the calli and their capacity for growth, *P. aegyptiaca *calli were dissected into pieces containing roots and placed in contact with roots of a tomato host in a minirhizotron system (Figure 3G). Eight days after *P. aegyptiaca *inoculation on tomato roots, the YFP-fluorescing haustorium penetrated the tomato root. Where haustoria entered the host root in the plane of the photo it is possible to visualize the wedge-shaped haustoria through cells of the tomato tissue (Figures 3I and 3J). In this case haustoria penetrated the host root simultaneously from lower left and upper right, providing a view of live haustoria. During the process of host parasitism the YFP marker also served as in indicator of tissue viability, whereby regions of the parasite explant that senesced and turned black as seen under white light also lost fluorescence under UV illumination (Figures 5A to 5F). Parasite tissues that formed successfull haustoria, in contrast, retained fluorescence and their development was clear when viewed over time. Twenty days after tomato inoculation 10 ± 0.0% of YFP-positive explants from each of the clones formed YFP-positive tubercles on the host (Figures 5E and 5F), and 5 days later secondary roots expressing YFP were fully developed in all of them (Figures 5G and 5H). One month after inoculation 83 ± 17% of YFP-positive "spider-like" stage tubercles developed YFP-positive shoot buds (Figures 5I and 5J). No significant differences in the tomato root infection efficiency and dynamics were observed between YFP-positive explants and YFP-negative controls (data not shown).

The methodology described here presents a robust system for generating and evaluating *Phelipanche *transformants. First, the system is efficient in producing large numbers of parasite calli for both the *Agrobacterium*-mediated transformation step and the multiplication of transformed calli for experimental evaluation (e.g., Figures 3G and 3H). A principle advantage is that a single cultured tubercle can produce enough explants for a transformation experiment and such genetic uniformity can be important when studying a genetically variable organism such as *P. aegyptiaca *[24]. Clonal propagation is also advantageous for multiplying transformed tissue because it ensures that even a single transformation event can be subdivided to produce sufficient numbers of transformed calli to satisfy experimental requirements. For example replication can be achieved at all levels by using more than one transformation event created from the same *A. rhizogenes *inoculation and then clonally propagating each event to reach any level of replication need for statistical analysis.

With respect to evaluating transgenic plants, the infective calli produced by this method are suitable for addressing many of the key questions in parasitic plant biology. Chief among these are questions that revolve around the haustorium and its initiation, development, and function. Other questions relating to post attachment development such as how tubercle growth is regulated and how roots and shoots develop are equally tractable. In contrast, questions relating to aspects of parasite seed development and germination will require completing the life cycle of the parasite. Although we have not described it here, production of transgenic *P. aegyptiaca *seed should be possible as this species is partially autogamous and can set seed under greenhouse or growth chamber conditions. During pilot studies we have successfully transplanted hosts and attached parasites to soil and observed development of a normal floral shoot (data not shown). While we plan to produce transgenic seed for future experiments, the currently reported method is analogous to established methods of studying transgenic facultative parasites [12-14] or using chimeric plants with transformed roots [25,26]. The transgenic *P. aegyptiaca *we describe are not chimeric, but are similar to products of other *A. rhizogenes *transformation protocols in being useful research materials that do not require the extra time for sexual reproduction. For *P. aegyptiaca *the time required from *A. rhizogenes *inoculation to having transgenic calli ready for evaluation on a host is approximately four months.

## Conclusions

Obligate parasitic plants are interesting examples of plant evolution and can be important agricultural pests [10]. There is increasing interest in understanding how these plants have evolved and how crops can be made resistant to parasitism. However, research on obligate parasites has been hampered by the inherent challenges in genetically manipulating an organism that is completely dependent on a host plant. Here we describe a protocol for growing and clonally propagating *P. aegyptiaca *in culture such that tissue can be generated in sufficient quantity for transforming the parasite and studying the resultant transformants. The roots of the resulting transgenic calli are parasitically competent and can thus be used to study processes of haustorial initiation and function. The development of this method is timely in that it comes shortly after the first large scale release of expressed gene sequences from *P. aegyptiaca *by the Parasitic Plant Genome Project [9]. Thus, it is now possible to envision research that systematically silences *P. aegyptiaca *gene candidates for essential parasitism genes. Many questions remain and much more work is needed, but the advent of this relatively simple and robust transformation system should greatly accelerate progress in understanding obligate parasitic plants.

## Methods

### *P. aegyptiaca in vitro *culture

*P. aegyptiaca *seeds were collected in 2000 from multiple parasitic plants growing on tomato in the location of May Ami, Israel (seeds provided by Dr. D.M. Joel). Seeds were surface-sterilized by sequential immersions in 75% ethanol and 1% sodium hypochlorite followed by three rinses in sterile distilled water as described previously [27]. Sterilized seeds were spread on sterilized glass fiber filter paper (GFFP) moistened with sterile distilled water and placed inside 10 cm Petri dishes and incubated in the dark at 25/20°C for 7 days to allow the conditioning period needed to germinate. Conditioned *P. aegyptiaca *seeds were induced to germinate by applying the synthetic germination stimulant GR24 at 2 mg L^-1 ^[21]. One day after GR24 application seeds were removed from the GFFP using a 1 ml pipette and plated on full strength Murashige & Skoog medium (MS) supplemented with 3% (w/v) of sucrose and 0.8% (w/v) of agar (slightly modified from Ben-Hod et al [18]) with the pH adjusted to 5.75. *P. aegyptiaca *germination percentage was observed by counting the percentage of emerged radicles seven days after GR24 (Figure 1A). Thirty days after GR24 application, the percentage of *P. aegyptiaca *radicles that developed tubercle-like swelling was evaluated (Figure 1B) and these calli were transferred to 150 ml Erlenmeyer flasks containing MS medium as described before except without agar. Flasks were placed in the dark at 20°C in a shaker at 100 rpm for two months. The calli were transferred to fresh medium approximately every 20 days. No hormones were added.

### Plasmid and bacteria

The vector used in this work was pHG8-YFP modified to contain the mas2' promoter controlling the YFP reporter gene [14,28]. The vector pHG8-YFP was transformed into *A. rhizogenes *MSU440 by electroporation [22].

### *Phelipanche *transformation

Prior to transformation *A. rhizogenes *was grown for two days at 27°C in solid medium composed of peptone 5 g L^-1^, yeast extract 3 g L^-1^, and agar 8 g L^-1^, supplemented with spectinomycin (100 μg/μl) and acetosyringone (400 μM). A single colony was transferred to 3 ml of liquid medium containing the same components except for agar, and was allowed to grow for 16 hours at 27°C. After this incubation the *A. rhizogenes *was collected by centrifugation at 8,000 rpm for one minute at 4°C and the pellet resuspended in half strength MS (^1/2^X MS) medium without sucrose, at optical density of 0.2 at 600 nm. In a laminar flow hood, a three month-old *P. aegyptiaca *callus (Figure 1C) was immersed in a 10 cm Petri dish containing 15 ml of the *A. rhizogenes *suspension and chopped into approximately 100 small pieces, with each piece containing a few developing roots. The dish was incubated at room temperature for 20 minutes. Subsequently, the *P. aegyptiaca *explants were blotted against sterile filter paper and transferred to solid^1/2^X MS sucrose-free medium supplemented with acetosyringone 400 μM, where they were co-cultivated with *A. rhizogenes *in the dark at 20°C for 5 days (Figure 1D). *P. aegyptiaca *explants were then washed with sterile distilled water and transferred to 150 ml Erlenmeyer flasks (Figure 1E) containing full strength MS liquid medium with 3% of sucrose and supplemented with 300 mg L^-1 ^of the antibiotic timentin (SmithKline Beecham Pharmaceuticals) and 100 μg/ml kanamycin. Thirty-five days after transformation, putative transgenic tissue was identified by the presence of YFP sectors (Figures 3A and 3B) and transformation frequency was calculated as the number of *P. aegyptiaca *explants with at least one area of YFP expressing tissue, using Olympus SZX12 stereomicroscope with a GFP filter set (excitation 461 to 500 nm, detection > 510 nm) and YFP-positive explants returned to fresh full strength MS medium for another 55 days.

The YFP-positive sectors developed on the calli at 90 days after transformation (Figures 3B and 3C). To select for the transgenic tissue, YFP-negative tissue was manually removed by observing and photographing calli placed inside sterile Petri dishes under UV light and then cutting away YFP-negative tissue under sterile conditions in a laminar flow hood. Then the YFP-positive tissue was returned to fresh MS liquid media and the process of observing and cutting repeated several times over the next 20 days, until all negative tissue was removed. In addition, the growing YFP-positive calli were divided into pieces to create a collection of clones for each transformation event (Figures 3E and 3F). After 110 days from the time of *A. rhizogenes *transformation, the *P. aegyptiaca *calli were transferred to liquid medium in which the strength of MS salts and sucrose was reduced by half for 10 aditional days in order to promote root and haustorium organogenesis. As negative controls, *in vitro *grown *P. aegyptiaca *calli were used following the same procedure from *A. rhizogenes *inoculation to callus division, but using *A. rhizogenes *that did not carry pHG8-YFP.

### PCR analysis and Southern blot hybridization

To confirm further the integration of the YFP gene in the YFP-fluorescing tissue, genomic DNA was isolated from YFP-positive and YFP-negative calli using a DNeasy Plant Mini Kit (Qiagen). Two polymerase chain reaction (PCR) primers were designed to amplify a 1000 bp section of the mas-YFP promoter-reporter gene construct ([28], GenBank accession number: AY995145). A second set of PCR primers were designed to amplify the *P. aegyptiaca *18S gene as a positive control. Sequences from the related parasitic species *Orobanche fasciculata*, *Orobanche ludoviciana*, *Orobanche multiflora *and *Conopholis americana *(respective GenBank accession numbers: U59960.1, U59953.1, U59952 and U59954.1) were aligned using Muscle in the Geneius Pro 5.3.4 software. The more conserved regions were used to design a set of forward and reverse 18S primers. Each PCR reaction contained 5 ng of genomic DNA or plasmid DNA, 0.5 μM of each forward and reverse primers, 12.5 of 2× iProof Master Mix (BIO-RAD) and conditions used as described in the manufacturer's protocol. PCR products were separated by electrophoresis through a 1% agarose gel (Figure 4).

Approximately 10 μg of genomic DNA from each of three different *P. aegyptiaca *YFP-positive calli, and YFP-negative control calli were digested with 100 u of XbaI, EcoRV or EcoRI respectively at 37°C for 16 hours. 300 ng of the plasmid DNA pHG8-YFP was similarly digested with 40 u XbaI. DNA was separated on a 0.65% TBE agarose gel at 60 V for about 24 hours. The gel was stained and photographed and then treated with 0.25 M HCl for 25 minutes. The gel was treated with 0.5 M NaOH+1.5 M NaCl two times 25 minutes each followed by treatment with 0.5 M tris, pH8.0+1.5 M NaCl two times 25 minutes each. DNA was transferred to a nylon membrane Nytran Supercharge (Whatman) overnight using a 'TurboBlotter' and 10× SSC. The membrane was UV linked, trimmed and air-dried. The filter was prehybridized using 6× SSC, 5× Denhardt's solution, and 0.5% SDS at 68°C for 4 hours. The filter was hybridized using a^32^P-labeled 1000 bp probe specific to the mas-YFP sequence. The probe concentration was ~3.5 × 10^6 ^dpm/ml in the HYBE buffer (same as the prehybe buffer). The hybridization was carried out at 68°C for 19 hours. The filter was then washed in 2× SSC+0.1%SDS at 68°C with three buffer changes over a period of 60 minutes. The filter was autoradiographed for 19 hours with an intensifier screen at -80°C. Due to excess loading of the control lane 1 (plasmid pHG8-YFP), the filter was re-exposed for 1 hour and then the control lane was trimmed off and the remaining filter was re-exposed for 9 days.

### Demonstration of transgenic parasitic competence

In order to demonstrate parasitic competence of the transgenic calli, three YFP-positive clones from a single transformation event and three YFP-negative control clones were removed from the^1/2^X MS medium in which they were growing, washed with sterile distilled water and placed next to roots of six tomato plants that were 20 days old and growing in a mini-rhizotron system [29]. To increase the chance of tomato root infection, each *P. aegyptiaca *callus was again chopped in small pieces containing from 3 to 6 *P. aegyptiaca *roots of approximately 5 mm length, just before inoculation on tomato. Ten *P. aegyptiaca *explants per clone were placed in close contact with the tomato roots (Figures 3G and 3H, Figures 5A and 5B). Twenty days after inoculation of transgenic *P. aegyptiaca *explants on tomato roots, the percentage of *P. aegyptiaca *attachment to the host was scored as the percentage of inoculated explants that attached to the host and produced a tubercle out of the total number of explants inoculated per tomato plant (Figures 5E and 5F). Thirty days after *P. aegyptiaca *explants were inoculated onto tomato roots, the percentage of shoot formation was evaluated out of the total number of regenerated *P. aegyptiaca *tubercles (Figures 5I and 5J).

## Competing interests

The authors declare that they have no competing interests.

## Authors' contributions

MFA and JHW conceived the work and wrote the article. MFA designed and performed the experimental work except for plasmid contruction. JIY and PCGB designed and generated the pHG8-YFP plasmid. DR contributed to development of the culture system and, together with JIY critically edited the manuscript. All authors have read and approved the final manuscript.
